# Relationship between serum phosphate and mortality in critically ill children receiving continuous renal replacement therapy

**DOI:** 10.3389/fped.2023.1129156

**Published:** 2023-04-12

**Authors:** Xiong Zhou, Jie He, Desheng Zhu, Zhenya Yao, Dan Peng, Xinping Zhang

**Affiliations:** Pediatric Department of Critical Care Medicine, Hunan Children’s Hospital, Changsha, China

**Keywords:** continuous renal replacement therapy, children, phosphate, mortality, pediatric intensive care unit

## Abstract

**Purpose:**

We aimed to explore the relationship between serum phosphate concentration and 90-day mortality in critically ill children receiving continuous renal replacement therapy (CRRT).

**Methods:**

Data from the medical records of children aged <13 years who received CRRT at the Pediatric Intensive Care Unit of Hunan Children's Hospital, China from January 2015 to June 2020 were retrospectively collected. Children were grouped into four categories according to the baseline phosphate concentration before CRRT and mean serum phosphate concentration during CRRT: <0.81 mmol/L (hypophosphatemia), 0.81–1.19 mmol/L, 1.2–2.4 mmol/L (normal phosphate concentration), and >2.4 mmol/L (hyperphosphatemia), with the normal phosphate group serving as the comparator group. The correlation of the serum phosphate concentration before and during CRRT with the 90-day mortality after CRRT initiation was analyzed using logistic regression.

**Results:**

A total of 177 children were included in our study. The mean serum phosphate concentration before CRRT was 1.46 mmol/L (quartiles: 1.04, 2.20). The 90-day mortality rate was increased in children with a serum phosphate concentration >2.4 mmol/L before CRRT (adjusted odds ratio [aOR] 3.74, 95% confidence interval [CI] 1.42–9.86, *P* = 0.008). The mean serum phosphate concentration during CRRT was 1.2 mmol/L (quartiles: 0.91, 1.49). The 90-day mortality rate was increased in children with a mean serum phosphate concentration >2.4 mmol/L during CRRT (aOR 7.34, 95% CI 1.59–33.88, *P* = 0.011).

**Conclusion:**

Hyperphosphatemia before and during CRRT predicts a higher 90-day mortality rate.

## 1. Introduction

Although serum phosphate accounts for only approximately 1% of phosphorus in the body, it plays an important role in energy metabolism, bone metabolism, cellular signal transduction, and oxygen transport (1–3). Currently, the generally accepted normal range of serum phosphate in adults is 0.8–1.5 mmol/L (2.5–4.5 mg/dl) (4). Phosphate homeostasis is a complex process, and its abnormalities can cause dysfunction of multiple organ systems, including the cardiovascular, respiratory, immune, neuromuscular, and hematologic systems (5).

Phosphate concentration is typically abnormal in critically ill patients. There have been an increasing number of studies on the relationship between phosphate abnormalities and clinical outcomes (6–9). A recent large-scale meta-analysis showed that hyperphosphatemia is associated with all-cause mortality in critically ill patients (10). Additionally, some studies have shown that hypophosphatemia is an independent risk factor for morbidity and mortality (11, 12), whereas other reports have shown no significant association with either (6, 13). Therefore, it remains unclear whether an abnormal serum phosphate concentration is directly associated with increased mortality or is merely a marker of disease severity, in the context of intensive care unit (ICU) patients.

Phosphate, a small inorganic solute, is removed through diffusion or convection during continuous renal replacement therapy (CRRT). Patients receiving CRRT have an increased risk of developing hypophosphatemia, and this has been associated with poor prognosis (4, 14). Patients may require phosphate supplementation after experiencing hypophosphatemia; however, inappropriate supplementation may trigger hyperphosphatemia. Furthermore, large magnitude changes in the serum phosphate concentration have been associated with an increased in-hospital mortality rate (9). One of the goals of CRRT is correcting electrolyte disturbances; nevertheless, in some patients, phosphate abnormalities persist during CRRT. Patients requiring CRRT are often the most severely ill patients in the ICU—the mortality rate of these patients in the pediatric ICU (PICU) is more than eight times that of the total PICU population (15). Currently, only a limited number of studies have probed the effect of serum phosphate concentration on patient mortality in CRRT cases. In particular, there is no information available regarding the relationship between serum phosphate concentrations (before and during CRRT) and mortality in children. Therefore, this study aimed to assess the relationship between serum phosphate concentration before and after CRRT initiation with the 90-day mortality rate in critically ill children.

## 2. Material and methods

### 2.1. Study population

Data from the medical records of children who underwent CRRT in the Department of Critical Care Medicine at Hunan Children's Hospital from January 2015 to June 2020 were retrospectively collected. The normal serum phosphate concentration of children fluctuates slightly with age, whereas the reference value for children aged >13 years fluctuates greatly. Therefore, to facilitate group statistics, children >13 years of age were excluded from this study (4, 16). The inclusion criteria were as follows: (1) serum phosphate test results available within 24 h prior to CRRT initiation; (2) at least one serum phosphate test result during CRRT; and (3) a CRRT duration ≥24 h. The exclusion criteria were as follows: (1) mortality within 48 h of CRRT initiation; (2) oncologic disease; and (3) chronic kidney disease (17). If a child received multiple courses of CRRT while in the PICU, only the first course was included in our analysis.

This study was conducted with approval from the Ethics Committee of Hunan Children's Hospital (Approval Number: HCHLL-2022-150). This was a retrospective observational study; data were acquired from the electronic case records database; this research did not involve aspects related to patient privacy; and there was no commercial interest involved. Thus, the ethics committee waived the requirement for informed patient consent.

### 2.2. CRRT protocol

The equipment used for CRRT at our center was the multiFiltrate system (Fresenius Medical Care, Bad Homburg, Germany) or PRISMAflex System (Gambro, Lund, Sweden), including the ancillary tubing and filters. For vascular access, a double-lumen central venous catheter was inserted in the femoral or internal jugular vein. The treatment mode was continuous veno-venous hemodialysis or hemodiafiltration, heparin or sodium citrate was the anticoagulation agent, and phosphate-free replacement and dialysis fluids were used. Electrolytes were routinely monitored, every 6–8 h, and vital signs, hemodynamics, and coagulation were closely monitored throughout the CRRT treatment process. CRRT treatment parameters were titrated, as needed.

### 2.3. Data collection

Clinical and biochemical parameters that were recorded included sex, age (months), weight, Pediatric Critical Illness Score (18), whether or not mechanical ventilation was used, whether or not vasopressors were used, serum creatinine, albumin, sodium, potassium, and calcium concentrations, blood pH, and the blood platelet count.

The main predictive factors in this study were as follows: (1) serum phosphate concentration before CRRT initiation and (2) mean serum phosphate concentration during CRRT. The serum phosphate concentration before CRRT initiation was defined as the test result closest and within 24 h prior to CRRT initiation. The primary outcome was the 90-day mortality rate after CRRT initiation.

The range of normal serum phosphate concentration in children under 13 years of age fluctuated slightly with age, and the range from the lower limit of reference value 1.2 mmol/L (3.7 mg/dl) to the upper limit 2.4 mmol/L (7.4 mg/dl) was considered the range of normal serum phosphate concentration in these children (16, 19). Currently, there is no unified reference value for hypophosphatemia in children (8). In adult studies, hypophosphatemia has been defined as a serum phosphate concentration < 0.81 mmol/L (2.5 mg/dl) (4). Therefore, in this study, we have also used serum phosphate concentration < 0.81 mmol/L to define hypophosphatemia. Furthermore, there is also no clear standard for hyperphosphatemia. Nevertheless, we defined hyperphosphatemia as a serum phosphate concentration > 2.4 mmol/L.

When the serum phosphate concentration was <0.81 mmol/L, 0.5–1 ml/kg/session sodium glycerophosphate was empirically administered *via* slow intravenous infusion, with a maximum of 10 ml/session, and the decision to reintroduce intravenous phosphate was made according to the serum phosphate concentration.

Participants were divided into four groups according to their serum phosphate concentration: <0.81 mmol/L, 0.81–1.19 mmol/L, 1.2–2.4 mmol/L, and >2.4 mmol/L, with the 1.2–2.4 mmol/L group—the normal range for serum phosphate in children—set as the comparator group.

### 2.4. Statistical analysis

Data were analyzed using IBM SPSS Statistics, version 25.0 (IBM Corp., Armonk, NY). Data with a normal distribution are expressed as mean ± standard deviation ($\bar{x}\pm s$), and analysis of variance was used for comparisons between groups. Count data are expressed as number of cases (percentage), and comparisons were performed using the $\chi^{2}$ test. Measurement data with a non-normal distribution are expressed as median (quartiles) [*M*(*P25, P75*)], and the Kruskal–Wallis *H* test was used for comparisons between groups.

Logistic regression was used to analyze the odds ratios (OR) of the relationship between the serum phosphate concentration and the 90-day mortality rate. Confounding factors were controlled by adjusting for clinically relevant variables, including sex, age (in months), weight, Pediatric Critical Illness Score, whether or not mechanical ventilation was used, whether or not vasopressors were used, serum creatinine, albumin, sodium, potassium, and calcium concentrations, blood pH, and blood platelet count. Covariables were selected by force entry. Four-node restricted cubic splines were constructed to study the nonlinear relationship of the serum phosphate concentration before (baseline) and during CRRT with the 90-day mortality rate.

Differences were considered statistically significant at *P* < 0.05. Regression analyses and model constructions were performed using R-studio, version 4.2.1 (Integrated Development for R. RStudio, Inc., Boston, Massachusetts, USA); glm was used for the logistic regression, and rms was used for the restricted cubic spline curves.

## 3. Results

### 3.1. Baseline characteristics of participants before CRRT initiation

**Figure 1:**
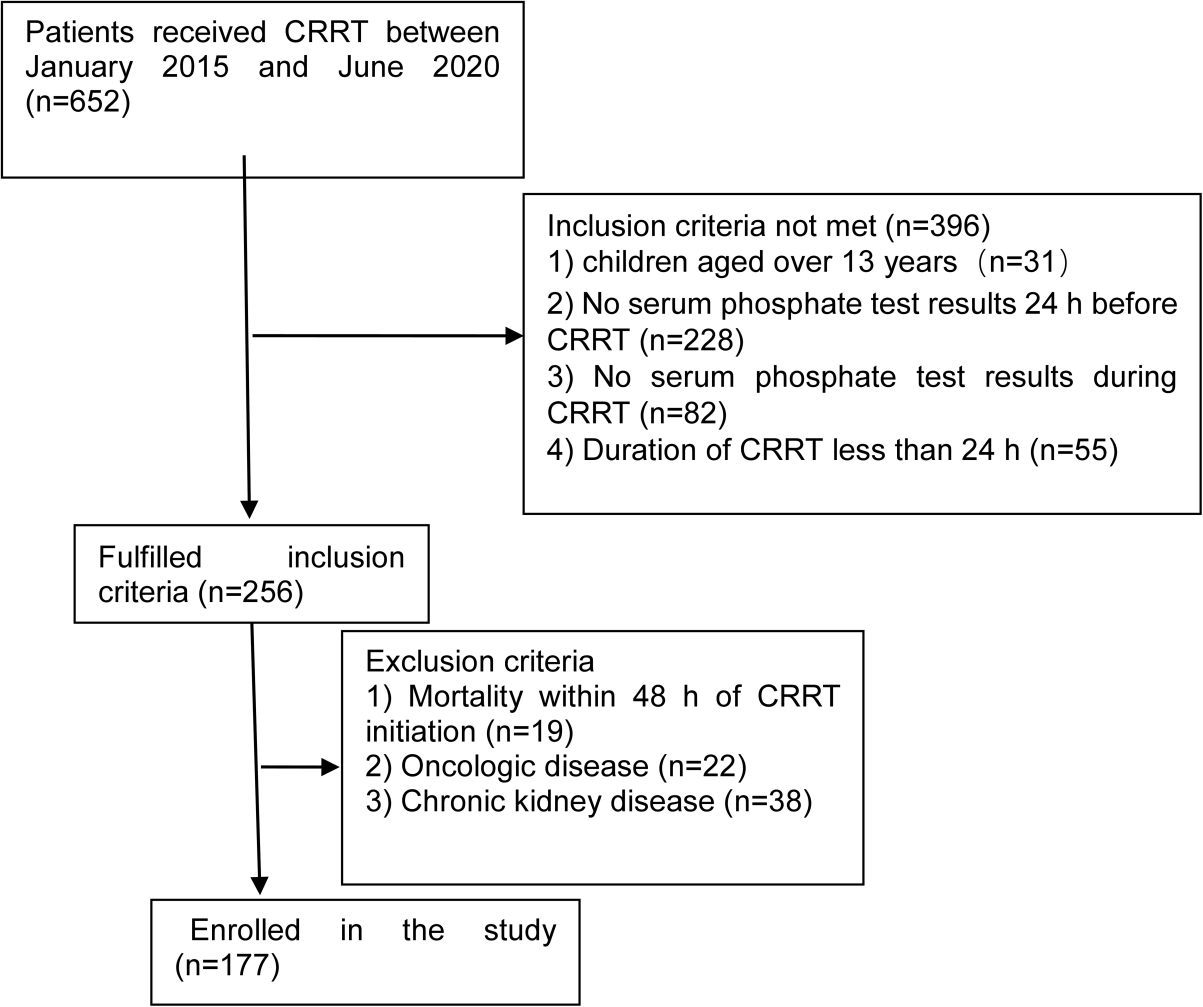
Flow chart showing participant categorization and interventions for this study. CRRT, continuous renal replacement therapy.

In total, 177 children were included in this study; details are shown in Figure 1. The male-to-female ratio was 112/65 (males, 63.3%). The median age was 27 months (quartiles: 9, 67), and the median weight was 12.4 kg (quartiles: 8, 20). The most common indication for CRRT was sepsis (35.6%). The median Pediatric Critical Illness Score was 78 (quartiles: 74, 80); 45.8% of participants (*n* = 81) required mechanical ventilation and 13.6% (*n* = 24) required vasopressors (Table 1).

**Table 1:** Baseline characteristics of the participants before CRRT initiation.

Parameter	Total	Serum phosphate concentration before CRRT (mmol/L)				*H*/$\chi^{2}$/*F-*value	*P*-value
		<0.81	0.81–1.19	1.2–2.4	>2.4		
Number of cases	177	18	42	79	38		
Sex (male/female)	112/65	10/8	26/16	51/28	25/13	0.655	0.884
Age [*M* (P25, P75), months]	27 (9, 67)	17.5 (10, 46.3)	27.0 (10, 98.8)	31 (9, 73)	24.5 (7.5, 66)	1.499	0.683
Weight [*M* (P25, P75), kg]	12.4 (8, 20.0)	11.9 (7.9, 17.0)	13.1 (8.4, 27.1)	12.6 (7.8, 20.7)	11.9 (7.3, 19.8)	1.529	0.676
**CRRT indications [cases (%)]**							
AKI	50 (28.2)	5 (2.8)	13 (7.3)	24 (13.6)	8 (4.5)	1.301	0.729
Sepsis	63 (35.6)	5 (2.8)	12 (6.8)	29 (16.4)	17 (9.6)	2.812	0.422
Acute poisoning	20 (11.3)	3 (1.7)	5 (2.8)	9 (5.1)	3 (1.7)	1.175	0.780
Cytokine storm	26 (14.7)	3 (1.7)	4 (2.3)	12 (6.8)	7 (4.0)	1.467	0.690
Inherited metabolic crisis	7 (4.0)	1 (0.6)	3 (1.7)	2 (1.1)	1 (0.6)	2.196	0.551
Acute liver failure	11 (6.2)	1 (0.6)	5 (2.8)	3 (1.7)	2 (1.1)	3.036	0.347
PCIS [*M* (P25, P75), score]	78 (74, 80)	78 (71.5, 78)	78 (74, 80)	78 (76, 80)	77 (74, 80)	1.679	0.642
Mechanical ventilation [cases (%)]	81 (45.8)	7 (4.0)	20 (11.3)	36 (20.3)	18 (10.2)	3.390	0.335
Vasopressors [cases (%)]	24 (13.6)	5 (2.8)	4 (2.3)	11 (6.2)	4 (2.3)	3.469	0.325
Serum creatinine [*M* (P25, P75), μmol/L]	38.5 (23.0, 87.1)	37.1 (20.9, 115.0)	28.4 (19.6, 70.0)	41.3 (22.9, 84.5)	52.0 (30.3, 92)	7.057	0.070
Serum albumin ($\bar{x}\pm s$, g/L)	31.6 ± 5.7	30.7 ± 4.9	30.3 ± 4.1	32.3 ± 6.4	31.7 ± 6.0	1.379	0.251
Serum sodium ($\bar{x}\pm s$, mmol/L)	136.5 ± 8.1	135.0 ± 7.2	136.4 ± 6.0	137.6 ± 8.3	135.1 ± 9.7	0.767	0.514
Serum potassium [*M* (P25, P75), mmol/L]	4.17 (3.50, 5.19)	4.2 (3.5, 5.9)	4.0 (3.5, 5.5)	4.1 (3.5, 4.7)	4.6 (4.1, 5.6)	6.771	0.080
pH [*M* (P25, P75)]	7.40 (7.34, 7.445)	7.43 (7.27, 7.46)	7.40 (7.32, 7.42)	7.41 (7.34, 7.45)	7.37 (7.34, 7.43)	2.864	0.413
Blood platelets [*M* (P25, P75), ×10^9^/L]	161 (66, 273.5)	105 (47.3, 205)	139 (77.5, 312.5)	185 (66, 272)	178 (91, 271)	2.763	0.430
Serum calcium [*M* (P25, P75), mmol/L]	1.98 (1.78, 2.18)	2.02 (1.81, 2.19)	1.96 (1.87, 2.08)	2.02 (1.79, 2.23)	1.83 (1.61, 2.11)	7.042	0.071
Serum phosphate [*M* (P25, P75), mmol/L]	1.46 (1.04, 2.20)	0.70 (0.63, 0.77)	0.99 (0.89, 1.08)	1.62 (1.37, 1.95)	2.84 (2.67, 3.27)	156.093	<0.001

CRRT, continuous renal replacement therapy; AKI, acute kidney injury; PCIS, pediatric critical illness score.

### 3.2. Serum phosphate concentration and 90-day mortality rate before CRRT initiation

**Figure 2:**
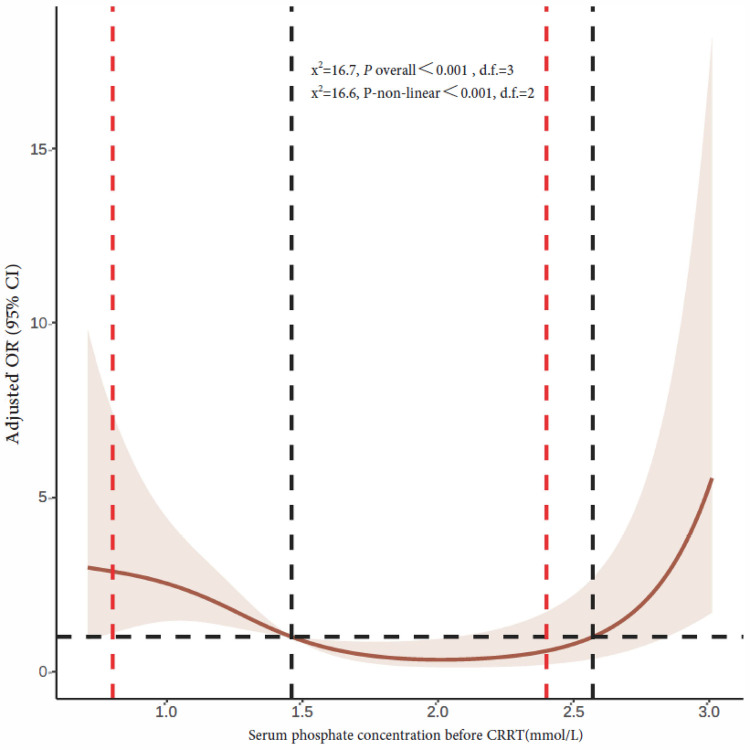
Restricted cubic splines of the correlation between serum phosphate concentration and 90-day mortality rate prior to CRRT initiation. The dotted red line on the left indicates hypophosphatemia cutoff, while the dotted red line on the right indicates hyperphosphatemia cutoff. The horizontal black dashed line represents OR = 1, and the vertical black dashed line is the serum phosphate concentration at the point where the curve intersects with OR value 1 that are 1.46 and 2.57 mmol/L, respectively. CRRT, continuous renal replacement therapy; OR, odds ratio; CI, confidence interval.

The mean serum phosphate concentration before CRRT initiation was 1.46 mmol/L (quartiles: 1.04, 2.20). The 90-day mortality rates of children with serum phosphate concentrations <0.81, 0.81–1.19, 1.2–2.4, and >2.4 mmol/L before CRRT were 50%, 35.7%, 26.6%, and 55.3%, respectively. Multivariate logistic regression analysis showed a higher 90-day mortality rate in children with a serum phosphate concentration >2.4 mmol/L before CRRT than in children with a serum phosphate concentration of 1.2–2.4 mmol/L [adjusted OR 3.74, confidence interval (CI) 1.42–9.86, *P* = 0.008]. Serum phosphate concentrations <0.81 and 0.81–1.19 mmol/L were not associated with significant increases in the 90-day mortality rate (Table 2). Figure 2 shows restricted cubic splines for the adjusted OR of the serum phosphate concentration before CRRT and the corresponding 90-day mortality rates.

**Table 2:** Relationship between serum phosphate concentration and 90-day mortality rate before CRRT initiation.

Serum phosphate concentration at CRRT initiation (mmol/L)	Number of cases	90-day mortality rate (cases/%)	Single-factor analysis		Multifactor analysis	
			OR (95% CI)	*P*-value	Adjusted OR (95% CI)*	*P*-value
<0.81	18	9/50	2.76 (0.97, 7.89)	0.058	2.87 (0.87, 9.46)	0.082
0.81–1.19	42	15/35.7	1.53 (0.69, 3.43)	0.297	1.82 (0.69, 4.8)	0.225
1.2–2.4	79	21/26.6	1 (ref)	1	1 (ref)	1
>2.4	38	21/55.3	3.41 (1.52, 7.68)	0.003	3.74 (1.42, 9.86)	0.008

CRRT, continuous renal replacement therapy; OR, odds ratio; CI, confidence interval.

* Adjusted for sex; age (in months); weight; Pediatric Critical Illness Score; whether the child was mechanically ventilated; whether vasopressors were used; serum creatinine, albumin, sodium, potassium, and calcium concentrations; blood pH; and platelet count.

### 3.3. Mean serum phosphate concentration and 90-day mortality rate during CRRT

**Figure 3:**
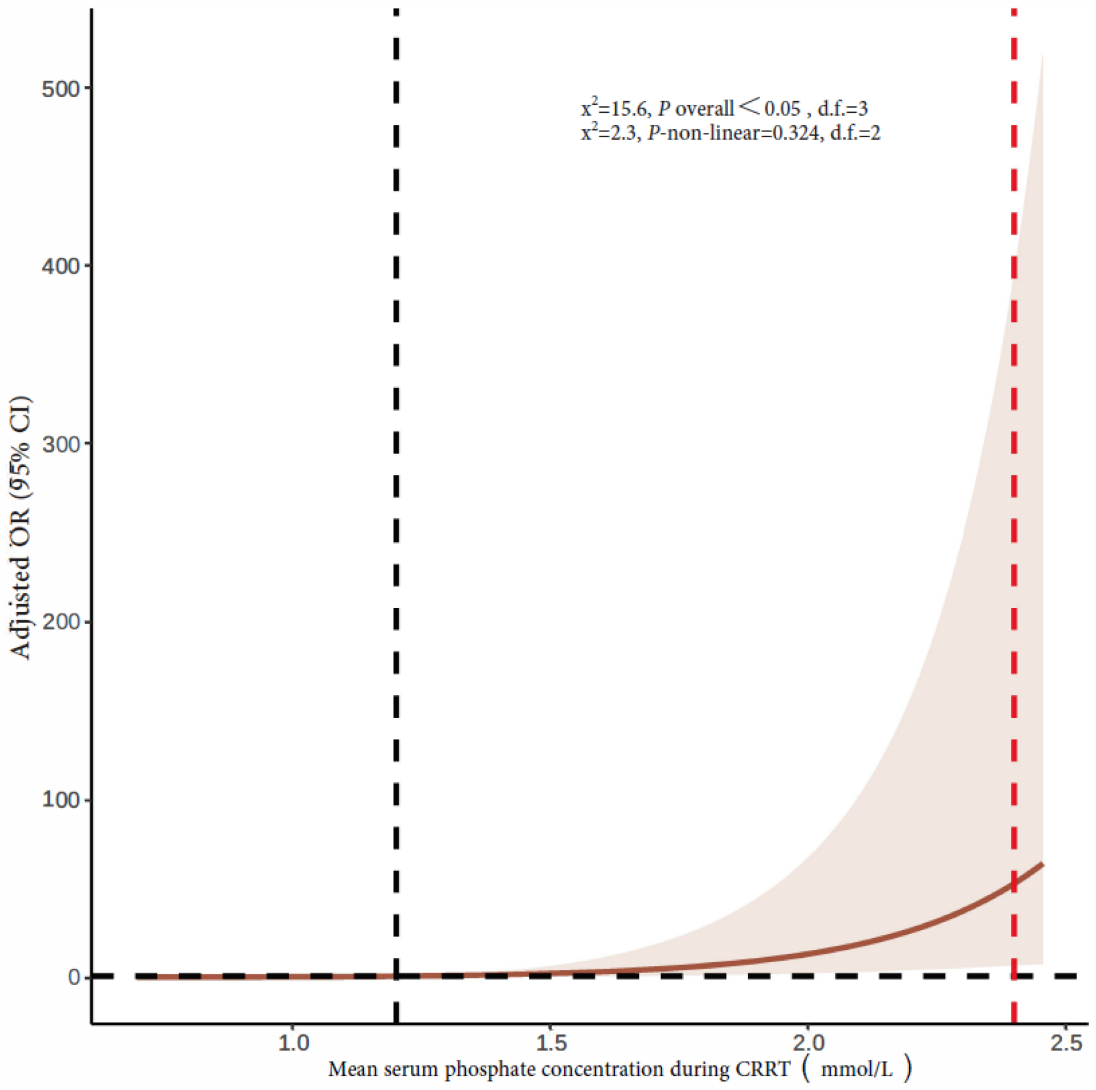
Restricted cubic splines of the association between mean serum phosphate concentration during CRRT and 90-day mortality rate. The vertical dotted red line indicates hyperphosphatemia cutoff, and the dotted black line indicates an OR of 1. CRRT, continuous renal replacement therapy; OR, odds ratio; CI, confidence interval.

The mean serum phosphate concentration during CRRT was 1.2 mmol/L (quartiles: 0.91, 1.49). The 90-day mortality rates of children with a mean serum phosphate concentration <0.81, 0.81–1.19, 1.2–2.4, and >2.4 mmol/L during CRRT were 34.5%, 32.2%, 33.3%, and 63.6%, respectively. Multivariate logistic regression analysis showed a higher 90-day mortality rate in children with a mean serum phosphate concentration >2.4 mmol/L during CRRT than in children with a serum phosphate concentration of 1.2–2.4 mmol/L (adjusted OR 7.34, 95% CI 1.59–33.88, *P* = 0.011). Hypophosphatemia during CRRT was not significantly correlated with mortality (Table 3). Figure 3 shows restricted cubic splines for the adjusted OR of the mean serum phosphate concentration during CRRT and the corresponding 90-day mortality rates.

**Table 3:** Relationship between mean serum phosphate concentration during CRRT and 90-day mortality rate.

Mean serum phosphate concentration during CRRT (mmol/L)	Number of cases	90-day mortality rate (cases/%)	Single-factor analysis		Multifactor analysis	
			OR (95% CI)	*P-*value	Adjusted OR* (95% CI)	*P-*value
<0.81	29	10/34.5	1.05 (0.42, 2.63)	0.912	0.53 (0.14, 2.08)	0.366
0.81–1.19	57	19/32.2	1 (0.4751, 2.1047)	1	0.78 (0.28, 2.2)	0.639
1.2–2.4	69	23/33.3	1 (ref)	1	1 (ref)	1
>2.4	22	14/63.6	3.5 (1.28, 9.54)	0.014	7.34 (1.59, 33.88)	0.011

CRRT, continuous renal replacement therapy; OR, odds ratio; CI, confidence interval.

* Adjusted for sex; age (in months); weight; Pediatric Critical Illness Score; whether the child was mechanically ventilated; whether vasopressors were used; serum creatinine, albumin, sodium, potassium, and calcium concentrations; blood pH; and platelet count.

## 4. Discussion

Phosphate, the most abundant intracellular anion in the body, is an important component of multiple physiological processes affecting many different organ systems. Some studies have correlated phosphate metabolism disturbances with prognosis in critically ill patients (6, 7, 10, 11, 20). However, few studies have focused on the relationship between serum phosphate concentration and clinical outcomes in patients receiving CRRT in the ICU (11, 21–24). In particular, the relationship between serum phosphate concentration and mortality in critically ill children undergoing CRRT in the PICU has not been reported. To our knowledge, this is the first cohort study to address this relationship.

A recent study by Thongprayoon et al. (21), conducted among adult ICU patients, showed that hyperphosphatemia was more common than hypo- and normophosphatemia before undergoing CRRT, patients with hyperphosphatemia could maintain normal serum phosphate levels during CRRT, and hyper- and hypophosphatemia before and during CRRT predicted a higher 90-day mortality rate. In our study, the baseline serum phosphate concentration before CRRT was usually within the normal range. Importantly, the included patients were from different types of ICUs, and patients in the hyperphosphatemia group had higher serum creatinine concentrations and lower glomerular filtration rates. However, in our study, patients were predominantly from the PICU, and there was no significant difference in the creatinine concentration before CRRT initiation between groups with different serum phosphate concentration ranges. The different sources of patients may explain the varied distribution of serum phosphate concentration in the Thongprayoon et al. study. In our study, patients with hyperphosphatemia before CRRT had a higher 90-day mortality rate than those with hypo- and normophosphatemia. Wang et al. (22) conducted a study among 796 patients with sepsis receiving CRRT and confirmed that when the serum phosphate concentration before CRRT was 5.6–8.7 mg/dl (1.8–2.8 mmol/L), patients had a significantly increased risk of 28-day mortality. Similar results were obtained in an observational study conducted among 1,144 critically ill patients with acute kidney injury who underwent CRRT (23); this study revealed that hyperphosphatemia during CRRT initiation was significantly associated with increased 28-day [hazard ratio (HR) 1.05, 95% CI 1.02–1.08, *P* = 0.001] and 90-day (HR 1.05, 95% CI 1.02–1.08, *P* = 0.001) mortality rates. In an earlier study, Thongprayoon et al. (21) also reported that hypophosphatemia before CRRT was associated with higher mortality rates. However, we have not observed an association of hypophosphatemia before CRRT with the 90-day mortality rate in our present study, and this is consistent with the results of two similar studies (23, 24); nevertheless, one of these studies has suggested that hypophosphatemia may prolong the length of stay in ICU for patients receiving CRRT (25). The relationship between hypophosphatemia before CRRT and prognosis varies between studies, and this variability may stem from differences in the study population, sample size, timing of CRRT intervention, and disease severity.

The present study found that the 90-day mortality rate increased when the mean serum phosphate concentration during CRRT was >2.4 mmol/L (adjusted OR 7.34, 95% CI 1.59–33.88, *P* = 0.011). This finding was consistent with those of several recent studies conducted among adults which have reported that hyperphosphatemia during CRRT predicts a higher risk of mortality (21–23). Wang et al. (22) also found that when the phosphate concentration was >3.8 mg/dl (1.2 mmol/L) 24 h after CRRT initiation, each 1 mg/dl increase raised the risk of 28-day mortality by 23% (adjusted OR 1.23, 95% CI 1.15–1.33). Patients with an elevated phosphate concentration during CRRT had significantly shorter survival times. Compared with patients with low phosphate concentrations, those with elevated phosphate concentrations had a 1.51-fold (95% CI 1.24–1.86, *P* < 0.001) and 1.50-fold (95% CI 1.24–1.82, *P* < 0.001) increased risk of 28-day and 90-day mortality, respectively (23).

Elevated phosphate concentrations increase fibroblast growth factor 23 (FGF23) levels, and this directly induces the *in vitro* hypertrophic growth of cardiomyocytes. High FGF23 concentrations are associated with left ventricular hypertrophy and cardiovascular toxicity, increasing the risk of adverse cardiovascular outcomes and ultimately leading to poor prognosis (26–28). Hyperphosphatemia causes vascular calcification *via* the downregulation of transcription factor EB in vascular smooth muscle cells. Meanwhile, microparticles from hyperphosphatemia-stimulated endothelial cells promote vascular calcification through astrocyte-elevated gene-1, and vascular calcification leads to a higher mortality rate (29, 30).

This study has some limitations. First, it is a single-center retrospective observational study, and there may be a potential for bias as well as confounding factors that could have affected our interpretation of the results. Second, the data were captured from an electronic medical records database and the system neither archived information on the causes of phosphate disturbances at baseline and during CRRT, nor denoted the causes of mortality. Therefore, the causal relationship between serum phosphate concentration and mortality remains unclear, and it is not possible to ascertain whether patient mortality was due to hyperphosphatemia or other underlying causes. Third, the efficacy of CRRT in phosphate removal varies with the effluent dose (24). We did not assess the actual effluent dose achieved during CRRT nor the effect of phosphate changes on mortality. Fourth, patients without baseline phosphate test results, those with undetectable phosphate levels during CRRT, and children older than 13 years were excluded—this may have potentially biased the results. Fifth, in our study, serum creatinine levels were included in the multifactorial analysis, while urine volume data were not collected or included in the analysis. Serum creatinine levels and urine volume have been reported to correlate with the severity of acute kidney injury, while serum phosphate levels have been shown to be dependent on renal function (31). Children in our study were grouped according to different phosphate levels, and there was no significant difference in baseline creatinine levels between the groups (*P* > 0.05). However, urine volume was not included in the multivariate analysis to take into account the effect of pRIFLE score on results, and this is indeed a limitation. In addition, according to standard practice, the ratio of the population to variables should be 10:1. However, in the present study, we did not select covariables through single-factor analysis, and all clinically relevant variables were included: this is a potential limitation of this study.

Phosphate is a potential biomarker of disease severity in critically ill patients receiving CRRT and is associated with a high mortality rate. Early identification and treatment of hyperphosphatemia is therefore important in patients receiving CRRT in the ICU. In future, we plan to conduct a randomized controlled trial that incorporates the phosphate concentration into the risk stratification and explores whether reducing the phosphate concentration, such as by CRRT, may reduce mortality risk in patients with hyperphosphatemia.

## Data Availability

The original contributions presented in the study are included in the article, further inquiries can be directed to the corresponding author.
